# Investigating the Corrosive Influence of Chloride Ions on Slag Recovery Machine Shells in Power Plants

**DOI:** 10.3390/ma16155270

**Published:** 2023-07-27

**Authors:** Yaohong Yu, Jintao Bai, Xiaohan Ma, Shengxing Wang, Dalong Hu, Jun Niu, Jiangtao Zhang, An Du, Dongqi Sun, Jian Lu, Yongzhe Fan

**Affiliations:** 1 Xi’an TPRI Water Management & Environment Protection Co., Ltd., Xi’an 710054, China; 2 School of Materials Science and Engineering, Hebei University of Technology, Tianjin 300130, China; 3 Key Lab for New Type of Functional Materials in Hebei Province, Tianjin 300130, China

**Keywords:** desulfurization wastewater, Q235-A steel, corrosion mechanism, corrosion products

## Abstract

An effective strategy for achieving cost-effective and environmentally friendly desulfurization wastewater in coal-fired power plants involves the incorporation of desulfurization wastewater into the slag water system. The objective of this study was to analyze the corrosion behavior of Q235-A slag-picker shell material upon the introduction of FGD wastewater into the slag water system. The dynamic weight loss method, electrochemical testing method and microscopic phase characterization were employed to investigate the impact of varying chloride ion concentrations (ranging from 1000 mg/L to 30,000 mg/L) of flue gas desulfurization wastewater (FGD wastewater) on the corrosion of Q235-A slag-picker shell material. The test results indicate that as the concentration of chloride ions increases, the corrosion rate increases from 1.1487 mm/a to 1.5590 mm/a when the concentration is less than 10,000 mg/L. However, when the concentration exceeds 10,000 mg/L, the corrosion rate decreases from 1.559 mm/a to 1.0393 mm/a. The corrosion rate is above 1 mm/a at all concentrations. As the Cl^−^ concentration, the quality of the corrosion product film initially increases and then decreases. The primary components of the corrosion product are α- FeOOH, γ-FeOOH, β-FeOOH, Fe_3_O_4_ and γ-Fe_2_O_3_.

## 1. Introduction

Desulfurization wastewater generated by coal-fired power plants poses significant challenges for treatment due to its complex composition, diverse pollutant types, and intricate remediation process. It represents the final stage of wastewater produced in power plants and requires specialized treatment approaches [1,2,3]. Currently, the treatment of desulfurization wastewater primarily involves two approaches: zero discharge treatment and standard discharge treatment. The zero discharge treatment integrates pretreatment softening, thick membrane water, and evaporation crystallization or flue evaporation technologies to eliminate desulfurization wastewater. Some power plants have adopted this process, which meets the treatment standards. However, this approach suffers from drawbacks such as a lengthy process route, substantial investment, and high-operational expenses. Utilizing desulfurization wastewater as make-up water for slag cooling, thereby enabling in-plant reuse of wastewater, offers several benefits, including cost-effectiveness, minimal investment and operational expenses, uncomplicated equipment requirements, and excellent adaptability. In contrast, the slag possesses the ability to absorb and eliminate suspended particles, certain heavy metals, and inorganic ions found in the wastewater. Additionally, when subjected to high temperatures, the slag can undergo partial evaporation, thereby reducing the volume of FGD wastewater introduced into the system.

Therefore, incorporating desulfurization wastewater into the slag water system as make-up water has emerged as a viable approach to mitigate the discharge of desulfurization wastewater, attracting growing interest in recent times [4,5,6]. The systematic treatment of power plant wastewater by introducing desulfurization wastewater into the slag water system has the advantages of treating waste with waste, low investment cost, and a simpler process modification [7,8,9,10,11]. Zhang et al. [12]. conducted a comprehensive analysis of three different FGD wastewater treatment technologies, examining their respective characteristics, treatment effectiveness, process parameter boundaries, potential impacts, and operational costs based on real-world engineering cases. Furthermore, they suggested that a comprehensive evaluation of the slag water system should be conducted after studying the integration of FGD wastewater into the system. Chen Biao et al. [13] studied the discharge of flue gas desulfurization wastewater from power plants into the slag water treatment system. The results showed that alkaline slag water could neutralize the desulfurization wastewater and cause the deposition of heavy metals and fluoride. Similarly, Yijin Zou [14] systematically examined the slag machine’s effect on the slag water system’s operation after introducing desulfurization wastewater into the slag water system. The results showed that the desulfurization wastewater introduced into the slag water system through the slag bin has a smaller impact on the overall system water quality. It is relatively small, mainly because all the operating parameters of the slag water system are within the range of stable operation. The discharge of the bottom discharge sludge of the high-efficiency thickener into the gypsum buffer tank will not affect the quality of gypsum. In contrast, the leaching toxicity of slag and gypsum meets the standard and the reuse is unaffected. However, the clogging and equipment corrosion of the slag water system piping, etc., by Cl^−^ in the desulfurization wastewater, requires further study, and its corrosion risk is not yet known.

The precise prediction of corrosion rate and understanding of corrosion mechanisms play a crucial role in selecting suitable equipment materials for protection. Underestimating the corrosion rate can result in catastrophic failures, environmental risks, and significant economic losses [15,16,17]. Therefore, studying the evolution and corrosion mechanisms of Q235-A steel in the sludge water system after the introduction of FGD wastewater can serve as a foundation for extending equipment lifespan and ensuring its integrity. The prediction of service life can provide a basis to ensure the safety of industrial production. The electrochemical behavior of Q235-A steel in a variety of corrosive media has been widely studied [18,19,20], such as Feng Wengui et al. [21], who analyzed the corrosion pattern and corrosion mechanism of Q235 carbon steel sheet in the cooling water of hydropower plant; and Lin Xiaofeng et al. [22] who studied the effect of temperature, pH, and conductivity on the corrosion of different components of the slag water system. However, the morphological evolution of the surface of Q235-A steel has rarely been monitored and analyzed, especially the lack of systematic research on the corrosion mechanism of Q235-A steel in the new environment.

Since the most significant change in the new environment after the addition of desulfurization wastewater to slag water is the Cl^−^ concentration, another important factor is because the Cl^−^ concentration in the water varies depending on the actual operation of the power plant. This means that the same fluid transfer equipment will work in locations with very different chloride concentrations. Industries, especially power plants, increasingly use recycled water to cope with scarcity constraints and sustainability needs [23,24,25,26,27,28]. The continuous water evaporation during the slag water cycle leads to changes in Cl^−^ concentrations; these recycled waters often have a higher salt content than when they were first used. Thus, it is clear [29,30,31,32,33] that understanding the effect of Cl^−^ concentration on the corrosion performance of engineering materials is worthy of in-depth studying.

In this paper, the corrosion rate of carbon steel surface under different concentrations of corrosion was determined by the corrosion hanging method with six different Cl^−^ concentration water samples; the microscopic morphology of corrosion product film and product composition were characterized by the surface analysis technique, and the change law of electrochemical corrosion parameters of carbon steel in cooling water was analyzed with the help of electrochemical measurement technique. Cl^−^ ions’ corrosion effect on the slag-fishing machine’s shell and its corrosion law was systematically studied.

## 2. Experimental

### 2.1. Material and Sample Preparation

Q235-A was chosen as the material for the experiments with the following compositions: C ≤ 0.17%, Mn ≤ 0.14%, Si ≤ 0.35%, S ≤ 0.035%, P ≤ 0.035%, and the balance of Fe. 10 mm × 2 mm specimens for electrochemical experiments, and 20 mm × 20 mm × 2 mm specimens for observation of corrosion morphology. All samples were washed in anhydrous ethanol, 10% NaOH solution and 20% HCl solution and distilled water in order to remove oil and rust from the surface, and dried in a drying oven for two hours. The specimens used to measure the weight loss were punched with a Φ ≤ 5 mm hole.

The actual water samples from power plants are complex in composition, containing elements such as Mg, P, S, Cl, Ca, etc. The ion concentration in the water samples is constantly changing with the operation of the equipment, which cannot be measured quantitatively, so this experiment will use the power plant to retrieve disposable water samples for the experimental original water samples whose chloride ion concentration is 5879.057753 mg/L, and by adding NaOH to adjust the pH of the water samples to 9 and adding NaCl. The water samples were adjusted to different concentrations, i.e., six different Cl^−^ concentrations of desulfurization wastewater solutions configured as 1000 mg/L, 3000 mg/L, 5000 mg/L, 10,000 mg/L, 20,000 mg/L, 30,000 mg/L. These concentrations, 1000 mg/L, 3000 mg/L, 5000 mg/L, 10,000 mg/L, 20,000 mg/L, 30,000 mg/L, were noted as L_0.1_, L_0.3_, L_0.5_, L_1_, L_2_, L_3_ for convenience. Table 1 shows the original water quality. The experimental water samples were varied on this basis for pH and Cl^−^ concentration.

### 2.2. Characterization Methods

#### 2.2.1. Hanging Piece Experiment

Fifteen groups of 50 mm × 25 mm × 5 mm specimens were weighed with an accuracy of 0.01 g. The numbers were recorded; nine groups of specimens were set up with three parallel specimens, each at 20,000 mg/L, 80 rpm, and 60 °C, respectively, for observing the change in weight loss rate over time. A daily data collection process was executed for a period of 9 days. Specimens from six groups were weighed and labeled L_0.1_, L_0.3_, L_0.5_, L_1_, L_2_, and L_3_, with three parallel specimens set for each group. After immersing the specimens in the solution, the corrosion duration was recorded starting after the solution temperature was raised to 60 °C. The extracted samples undergo water rinsing to eliminate specific corrosion contaminants. The residue adhered to the surface was subjected to ultrasonic cleaning for 25 min by employing 1000 mL of hydrochloric acid at 36% concentration (HCl, ρ = 1.19 g/mL), 20 g of antimony trioxide (Sb_2_O_3_), and 50 g of stannous chloride (SnCl_2_). The samples were then dried, weighed, and placed in a desiccator. A blank experiment was conducted for each set of experiments. The corrosion rate was calculated by Equation (1):

(1) $$R=\frac{\left(M_{1}-M_{2}-M_{3}\right)\times 8.76}{STD}\times 10^{7}$$

In the above equation: *R*—is the corrosion rate, mm/a; *M*_1_—is the mass of the specimen before the experiment, g; *M*_2_—is the mass of the specimen after the experiment, g; *M*_3_—is the mass difference between the blank specimen before and after pickling, g; *S*—is the total area of the specimen, cm^2^; *T*—is the test time, h; *D*—is the density of the material, kg/m^3^.

#### 2.2.2. Q235-A Surface Corrosion Analysis

A JSM-6510A scanning electron microscope (SEM) was used to observe the microscopic morphology and cross-sectional morphology of the corrosion product film; the elemental distribution of the corrosion product film was determined by an JED-2300 energy spectrometer (EDS); the corrosion product on the surface of the sample was analyzed by using a Smartlab 9 KW X-ray diffractometer (XRD): the power is 4 KW, the speed is 10°/min, and the test range is 10° to 90° under the parameters of the overall test of the sample to analyze its corrosion product phase composition.

#### 2.2.3. Electrochemical Testing

**Figure 1:**
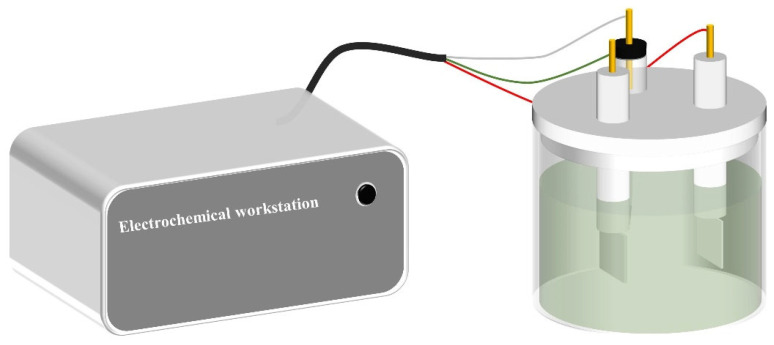
Schematic diagram of electrochemical test.

The electrochemical properties of the specimens were tested using the conventional three-electrode system, as shown in Figure 1. The experiment can obtain two kinds of data, impedance and polarization curve. Analyzing its impedance spectrum can determine its corrosion tendency and resistance size, and analyzing the polarization curve can derive the size of corrosion current density during the polarization process to analyze the corrosion process. The specimen has corrosion products as the working electrode, saturated glycury electrode (SCE) as the reference electrode, platinum electrode as the auxiliary electrode, a working electrode size of 10 mm × 10 mm × 2 mm, and an exposed working area of 1 cm^2^. EIS measurement under the OCP, low frequency 0.01 HZ, high frequency 100,000 HZ, amplitude 0.005; EIS test under each concentration. In order to analyze their corrosion product film changes, three experiments were conducted for 0 d, 3 d and 5 d, respectively; the results were fitted by Zsimpwin 3.60 software. All electrochemical experiments were conducted at 60 °C, and each experiment was repeated three times to ensure accurate results.

## 3. Results and Discussion

### 3.1. Hanging Experimental Analysis

**Figure 2:**
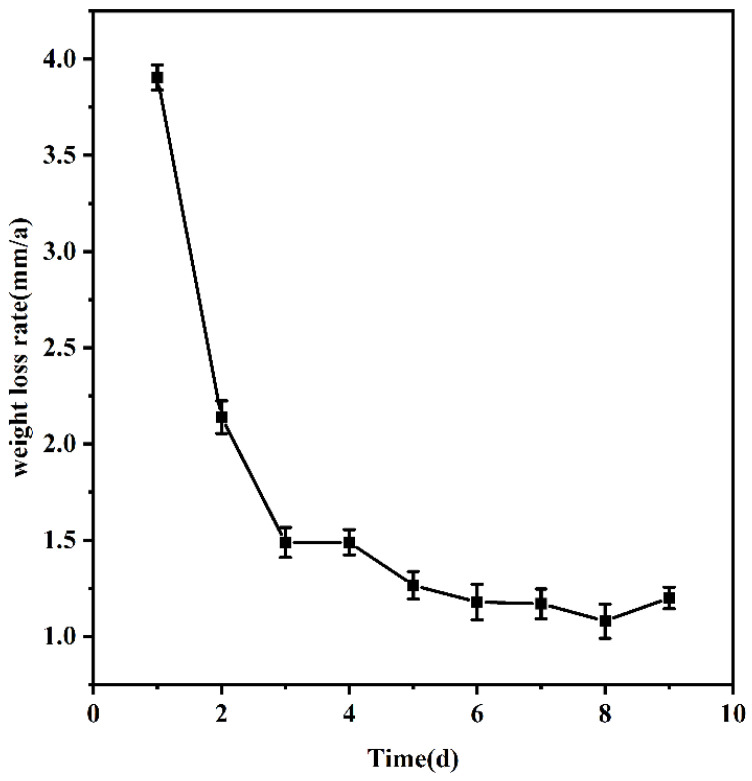
Weight loss rate curve with time.

It can be seen from Figure 2 that the Q235 steel in 20,000 mg/L solution corrosion rate gradually decreases with the extension of time. The corrosion rate achieves a state of gradual stability after approximately 5 days, ultimately stabilizing at a rate of 1.265 mm per annum. The accumulation of corrosion products on the surface of the sample impedes the continuation of corrosion over time. As time progresses, the production of corrosion products by the sample increases, gradually improving its structural integrity. This suggests that the corrosion products offer a degree of protection and stabilize the corrosion rate at a specific level [34]. The properties of the material and the concentration of chloride ions in the solution together determine the thickness of the corrosion product film. Therefore, when comparing the effect of chloride ion concentration on corrosion, the time should be fixed at 5 days.

**Figure 3:**
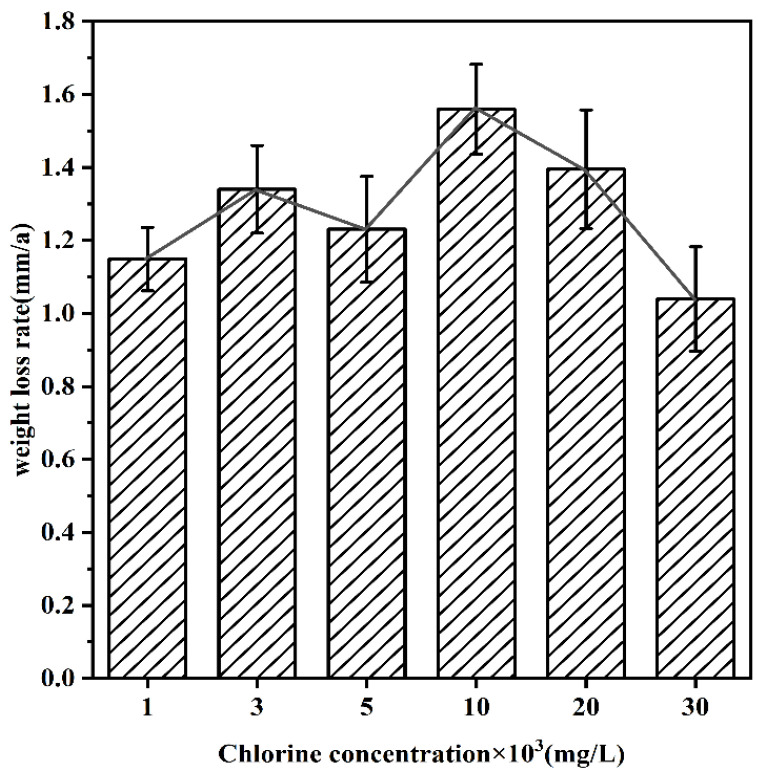
Weight loss rate of Q235-A in different Cl^−^ concentrations of FGD wastewater solution.

Figure 3 shows the weight loss rate of the samples in different concentrations of desulfurization wastewater. It can be found that the weight loss rate tends to increase with the increase of Cl^−^ concentration, reaching a peak at L_1_ and then decreasing with the increase of concentration, which is because the O content in the solution decreases with the increase of Cl^−^ concentration, making the weight loss rate decrease. It is noteworthy that the weight loss rate at L_0.5_ is lower than that at L_0.3_, probably due to the increase in Cl^−^ concentration and the adhesion of a water film on it. In addition, the water film hinders the occurrence of corrosion, resulting in a slight decrease in the weight loss rate.

### 3.2. Q235-A Surface Morphology Analysis

#### 3.2.1. Composition of Corrosion-Producing Films

**Figure 4:**
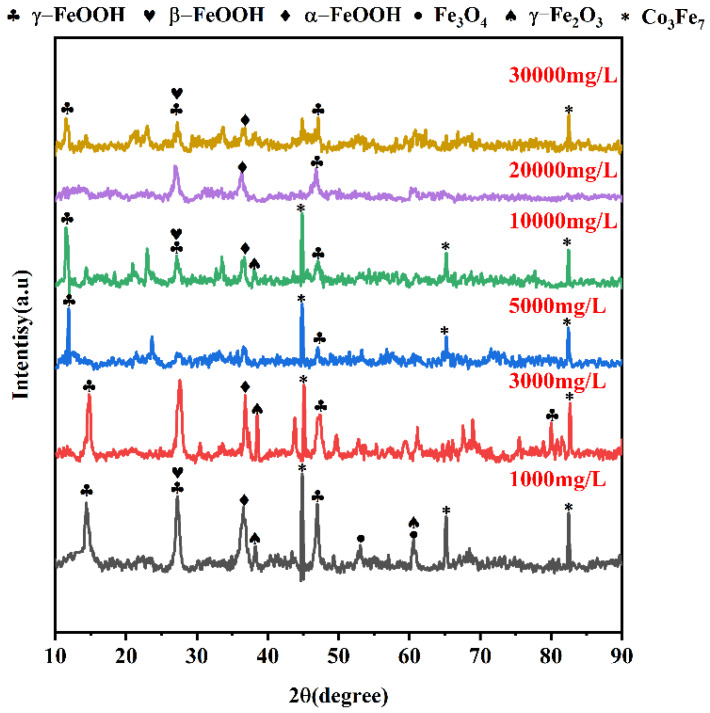
XRD results of corrosion product film of Q235-A steel.

To study the composition of corrosive products of Q235-A steel, the phase composition of the corrosion products was determined by X-ray diffraction after 5 days of corrosion in L_0.1_–L_3_ solution. As shown in Figure 4, the phase composition of the corrosion products of Q235-A in L_0.1_–L_3_ solution is the same, mainly composed of α-FeOOH, γ-FeOOH, β-FeOOH, Fe_3_O_4_ and γ-Fe_2_O_3_, which are similar to the corrosion products commonly found in the wet and salty marine atmospheric environment. A part of Co_3_Fe_4_ appears as the electron beam penetrates the sample to reach the Fe matrix. The Co_3_Fe_4_ phase gradually decreases with increasing Cl^−^ concentration, proving that the corrosion layer becomes more dense and complete. In addition, as the Cl^−^ concentration increases, the intensity of the α-FeOOH peak decreases, and the γ-FeOOH peak increase is indicative of Cl^−^ inhibiting the generation of α-FeOOH.

#### 3.2.2. Corrosion Product Form

**Figure 5:**
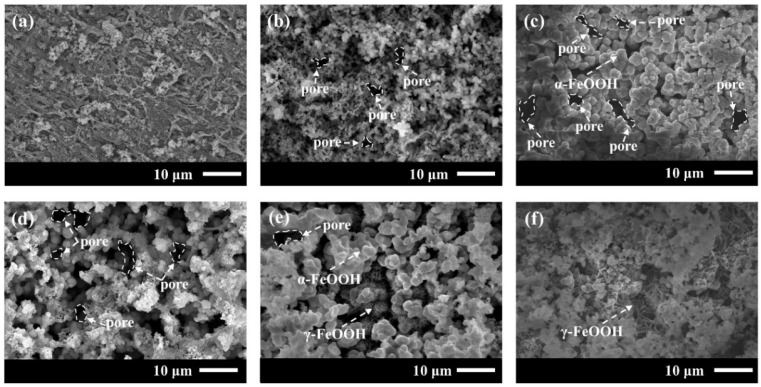
Microscopic morphology of Q235-A after 5 d corrosion in L_0.1_–L_3_ solution (**a**) L_0.1_, (**b**) L_0.3_, (**c**) L_0.5_, (**d**) L_1_, (**e**) L_2_, (**f**) L_3_.

To study the quality of Q235-A steel corrosion products, we observed the microscopic morphology of corrosion products of both steels after 5 d immersion, as shown in Figure 5. The Q235-A steel in L_0.1_–L_0.5_ solution after immersion corrosion products becomes granular, and there is no local corrosion and pitting; it is worth noting that in L_0.1_ corrosion products, film bonding is not strong. The corrosion products on it are very loose so it will expose part of the substrate. With the increase in Cl^−^ concentration, the corrosion product particles gradually become larger, and the denseness of the corrosion product film also increases because of the larger particles. However, after L_1_ gradually decreases because of the increase in pores, the L_1_ solution has larger holes in the corrosion product film. Pin-like corrosion products were produced in L_2_ and L_3_. According to the literature [35], α-FeOOH has a cotton ball-like and cluster structure, and γ-FeOOH has a needle band-like structure. Based on the phase of corrosion product and XRD results, it can be concluded that the rise with Cl^−^ will inhibit the conversion of γ-FeOOH to α-FeOOH.

**Figure 6:**
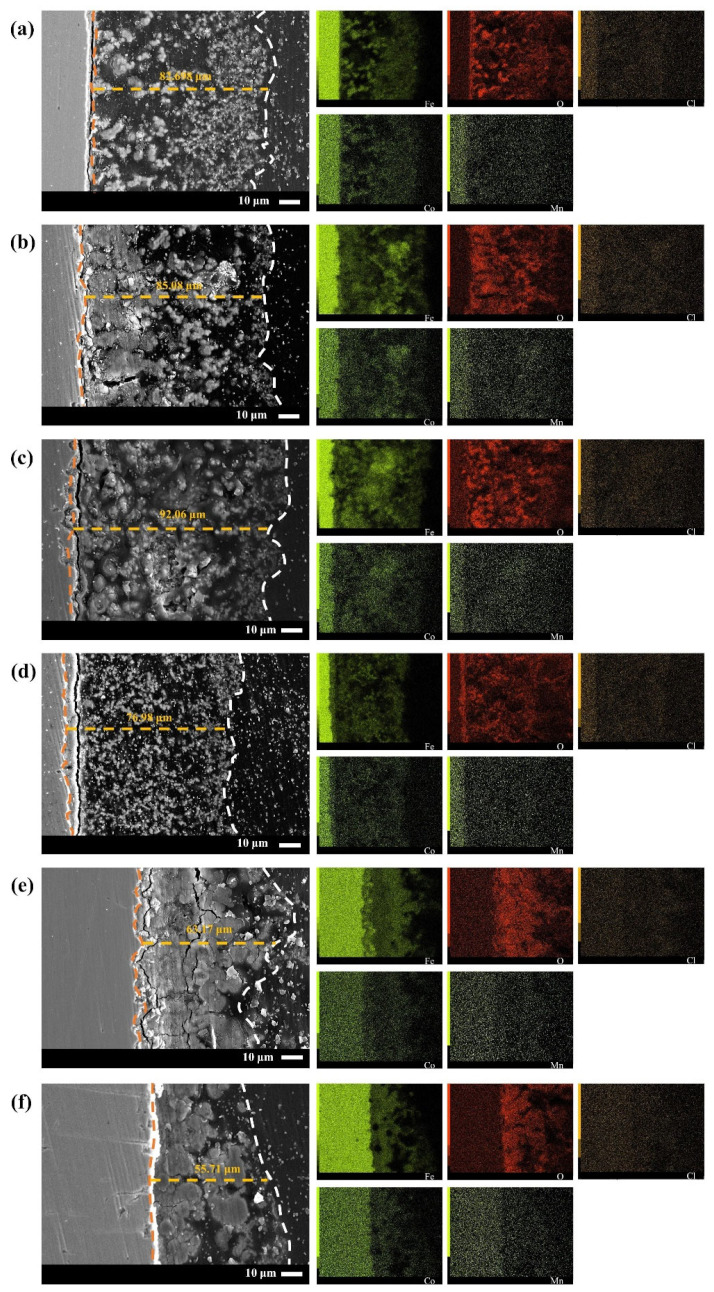
Cross-sectional morphology of Q235-A after 5 d corrosion in L_0.1_–L_3_ solution (**a**) L_0.1_, (**b**) L_0.3_, (**c**) L_0.5_, (**d**) L_1_, (**e**) L_2_, (**f**) L_3_.

The distribution of elements in the corrosion product film of Q235-A steel after 5 d of corrosion in L_0.1_–L_3_ solution was observed by SEM and EDS methods combined, as shown in Figure 6. As the increase of Cl^−^ concentration, the samples corrosion product film thickness first increases then decreases. The maximum is 92.06 μm, and the minimum is 55.71 μm. In addition, in L_0.1_ and L_1_, the outer film is loose and thick, while the inner membrane is thin, relatively dense, and tightly bound to the substrate. As the concentration increased, the inner film of corrosion products showed holes, large longitudinal and transverse cracks, and transverse and longitudinal cracks between the substrate and the inner film. The appearance of cracks is related to the orientation of the substrate surface, e.g., different gold structures are formed on different surface orientations. Cracks may start at the boundaries of the defective regions as well as in thin gold layers with concave edges formed on the initial Cu_3_Au substrate. Once cracks or initial pores begin to grow, they usually continue to grow in the same direction. It follows that cracks are largest on the (110) grain surface [36]. The metallographic organization of Q235-A steel after annealing, quenching, and tempering is tempered martensite, but there is no significant difference in the surface corrosion of grains with different surface energies due to the activated state of Q235-A steel. This indicates that Cl^−^ reduced the adhesion between the substrate and the inner film.

From the element distribution, Fe and O compounds are the main phases on the corrosion product film, which are consistent with the results of XRD. Most of the compounds are loose and porous; Cl elements are uniformly distributed on the corrosion product film, whereas Co elements and Mn elements are not enriched, indicating that they have no obvious protective effect on the substrate.

#### 3.2.3. Electrochemical Characteristics

**Figure 7:**
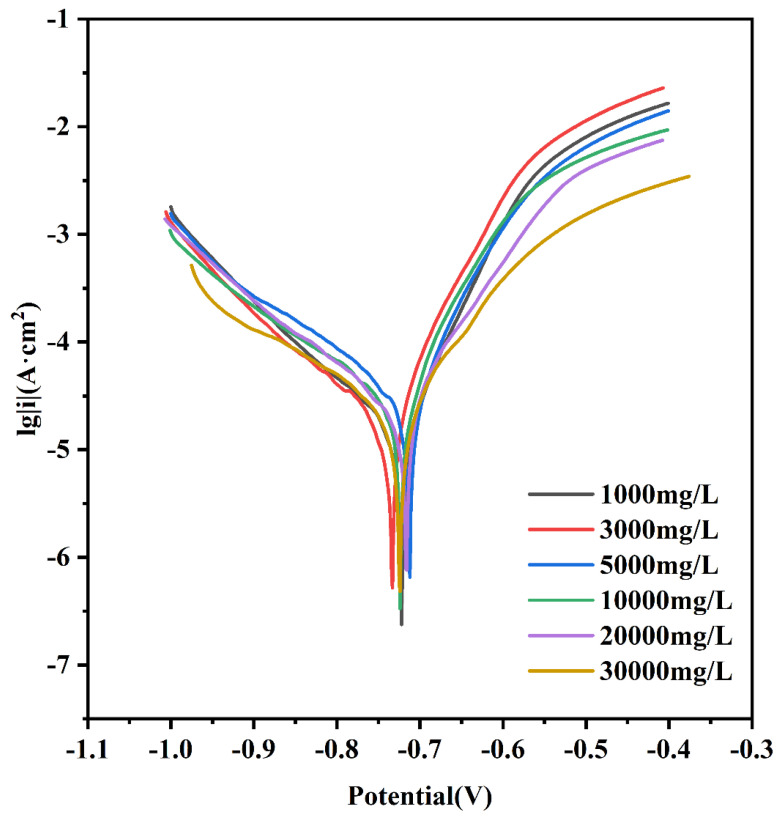
Dynamic potential polarization curve of Q235-A steel in L_0.1_–L_3_ solutio.

To determine the electrochemical properties of Q235-A steel in solution, the kinetic potential polarization curves in L_1_–L_3_ solutions were measured according to the experimental method described above (Figure 7). These curves show that the effect of solution concentration on the self-corrosion potential is not obvious, but L_0.1_ exhibits the lowest current density, which is related to the least amount of Cl^−^ in the solution, while L_1_ has the maximum current density, which decreases slightly as the concentration increases. This indicates that as the concentration increases, the reaction is inhibited and becomes more difficult. This is related to the decrease of O content in the solution and is consistent with the results obtained from the weight loss experiments. The corrosion potential and current density in each solution during the polarization test are shown in Table 2.

**Figure 8:**
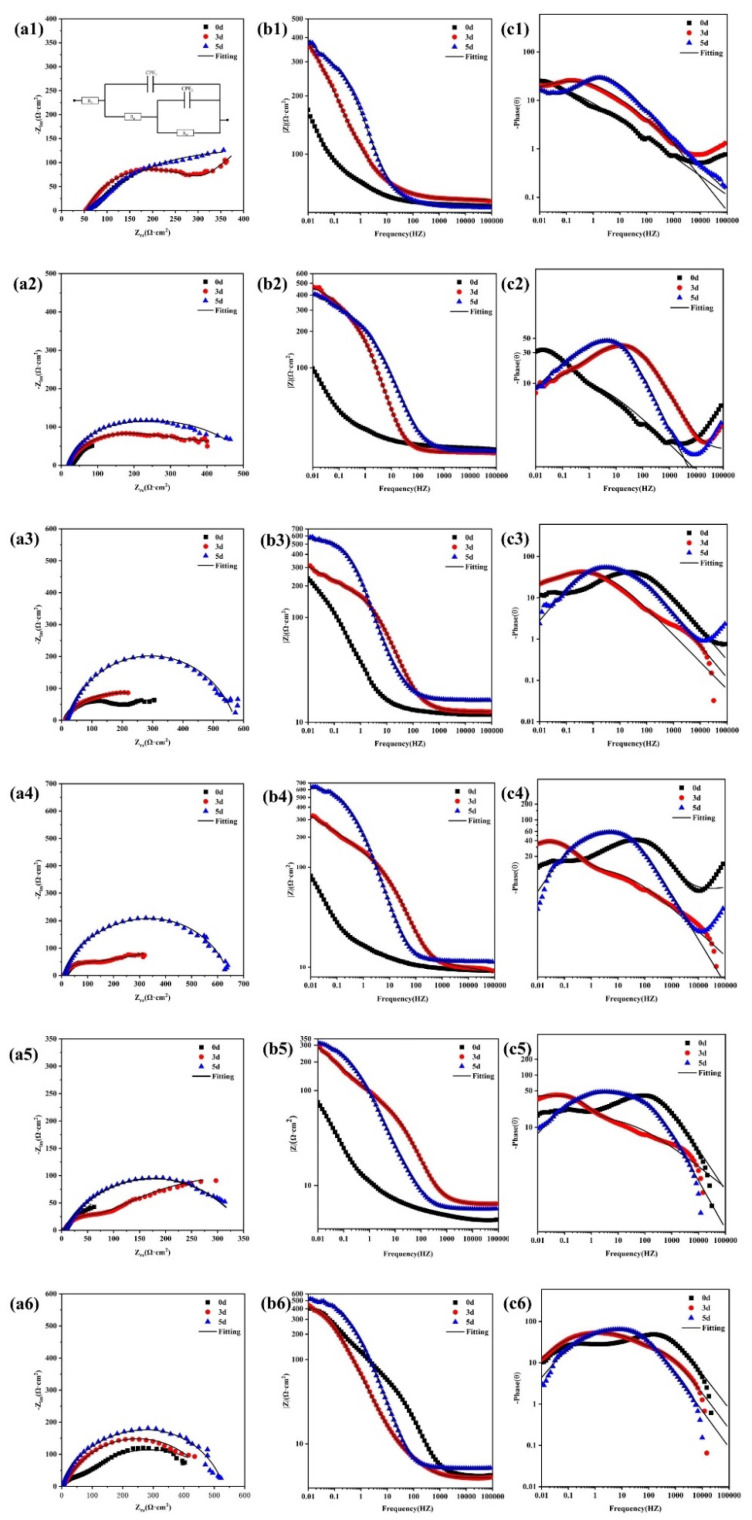
EIS results for Q235-A steel in L_0.1_–L_3_ solution with corrosion product film:L_0.1_ (**a1**–**c1**),L_0.3_ (**a2**–**c2**), L_0.5_ (**a3**–**c3**), L_1_ (**a4**–**c4**), L_2_ (**a5**–**c5**), L_3_ (**a6**–**c6**).

**Figure 9:**
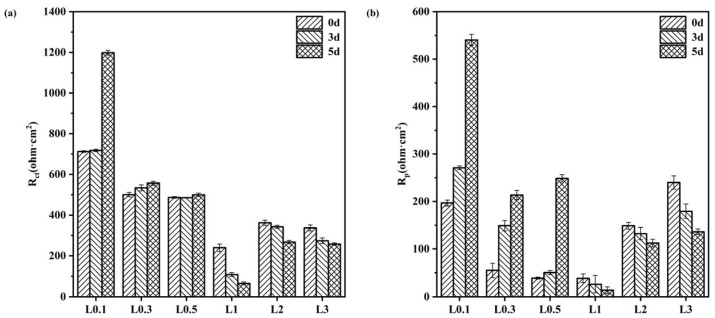
Rct (**a**) and Rp (**b**) of corrosion product film of Q235-A steel in L_0.1_–L_3_ solution.

Figure 8 shows the EIS results for Q235-A steel after 0 d, 3 d, and 5 d of corrosion in L_0.1_–L_3_ solutions, where a1-a6 are Nernst plots, b1–b6 are Bode absolute value plots, and c1–c6 are Bode phase angle plots. Table 3 shows the fitted results, and the Nyquist curve for Q235-A steel consists of an impedance arc. The Bode phase diagram shows two overlapping time constants: one represents the nature of the corrosion product film in the high-frequency region and the other time constant reacts with the charge transfer reaction at the film-electrolyte interface in the low-frequency region [37,38]. Based on the above analysis, the EIS curve was fitted with the equivalent circuit shown in Figure 8(a1) [39,40]. The equivalent circuit comprises two time constants, where R_s_ represents the solution resistance; Q_f_ represents the resistance of the corrosion product membrane; R_pore_ represents the pore-solution resistance of the membrane; Q_dl_ represents a dual electrode layer aconstant phase angle element; and R_ct_ represents the charge transfer resistance [41,42,43]. The values of R_ct_ and R_pore_ for Q235-A steel in the L_0.1_–L_3_ solution are shown in Figure 9. The values of R_ct_ and R_pore_ for Q235-A steel in L_0.1_–L_3_ solution are shown in Figure 9. L_0.5_ solution gradually increases, indicating that at low concentrations with time at R_ct_ and R_pore,_ both increase with time and its corrosion product film becomes denser, thus resistance is greater. And L_1_–L_3_ solution gradually decreases, indicating that at higher concentrations, with the increase of time, its corrosion product film becomes less dense resistant. At L_1_, R_ct_ and R_pore_ are the smallest. As Cl^−^ concentration increases, the corrosion product film protection of the substrate is same as the results of the hanging experiments with the trend of rising first and then declining.

## 4. Conclusions

From the results, corrosion with the extension of time in 5 d is gradually stable, in line with the corrosion mechanism that is free corrosion stage, corrosion control stage and stable corrosion stage. Free corrosion stage. In the initial stage of the metal surface in the activation state, the corrosion rate is very large, and shows an upward trend. The rust layer will be involved in the corrosion process, so this stage will not last long. Corrosion control stage. This stage is mainly due to the rust layer in the corrosion process from accelerated corrosion inhibiting the function of corrosion conversion, thus causing the corrosion rate reduction of the non-linear process. This process is quite complex, and there are many related theories (passivation film theory, microcell theory, etc.) to explain the role of the rust layer in the corrosion process. Stable corrosion stage. The corrosion rate eventually stabilizes the process. The function of the rust layer in the corrosion process has been determined, and the ultimate diffusion rate of dissolved oxygen in solution becomes the decisive factor affecting the corrosion rate. If other factors remain constant, corrosion will be carried out at this rate until the metal surface area changes dramatically.

With the increase of Cl^−^ concentration, the corrosion rate shows a trend of increasing and then decreasing, which in Cl^−^ concentration less than 10,000 mg/L, with the increase of Cl^−^ concentration, the corrosion rate increases, while in Cl^−^ concentration higher than 10,000 mg/L, the corrosion rate shows a decreasing trend; this is because with the increase of Cl^−^ concentration, O content will be reduced, thus hindering the corrosion process. In this paper, the corrosion mechanism of different concentrations of Cl^−^ in desulfurization wastewater on the slag-raking machine shell material was analyzed, and there is an inhibition efficiency relationship between its corrosion products and Cl^−^ concentration [44].

It is well known that Cl^−^ destroys the denseness of corrosion product films in tropical marine atmospheres with a higher Cl^−^ concentration, leading to the loosening of corrosion product films on steel surfaces [45]. The porosity of corrosion product films is determined by the size of FeOOH particles [46]. The pores between particles are positively correlated with FeOOH particles, and the nucleation and growth rate of FeOOH determines the size of FeOOH particles. The O content in the solution is negatively correlated with the Cl^−^ concentration, with the decrease of O content in the solution. Additionally, with the increase of Cl^−^ concentration, the decrease of other oxide content in the solution, due to anoxia, leads to the decrease of FeOOH nucleation sites, which makes the larger FeOOH generated in the corrosion product film. At lower concentrations, with the further increase of O concentration, more Fe_3_O_4_ is generated in the corrosion product, which makes the corrosion product film dense.

From the existing studies [47,48], it is known that, thermodynamically, the standard Gibbs free energy ΔGfθ of FeOOH (e.g., ΔGfθ = −495.748 kJ/mol for pinite α-FeOOH and ΔGfθ = −470.25 kJ/mol for fibrous iron ore γ-FeOOH) is higher than that of Fe_3_O_4_. ΔGfθ = −822.16 kJ/mol is higher [49]. Coupled with the fact that the experimental environment is liquid, it can be assumed that the Fe_3_O_4_ content becomes relatively high due to the difficulty of dehydrating the generated FeOOH to Fe_2_O_3_, which is then more easily transformed to Fe_3_O_4_, or due to the much more complicated process of transforming Fe^2+^ to FeOOH, resulting in the easy binding of Fe^2+^ to FeOOH on the desorbed surface of the specimen.

Combined with Figure 8 and Figure 9, it can be seen that at lower concentrations of corrosion products, film R_ct_ and R_p_ increase with time and become larger, indicating that the corrosion film gradually completes with time, but at higher concentrations; R_ct_ and R_p_ decrease with time, indicating that the denseness of its corrosion film becomes poor. In L_1_ solution, Q235-A steel R_ct_ and R_p_ are the smallest, combined with its corrosion products film interface diagram L_1_ when its corrosion rate reaches its highest. This further proves that, with the increase of Cl^−^ concentration, the solution of dissolved O decreases, which will lead to the redox reaction being more difficult to carry out. The original O position in the corrosion product film is occupied by Cl, making the structure of the corrosion product film become loose, thus exhibiting cracks of various sizes.

Corrosion products are α-FeOOH and Fe_3_O_4_ and a small amount of γ-FeOOH and Fe_2_O_3_; the corrosion mechanism under alkaline environment can be known from the following formula [50,51,52]:

Fe − 2e^−^ = Fe^2+^ (2)

Fe^2+^ + OH^−^ = Fe(OH)_2_ (3)

Fe(OH)_2_ + O_2_ = FeOOH (4)

FeOOH + Fe^2+^ = Fe_3_O_4_ (5)

(6) $$FeOOH\overset{Dehydration}{\to }{Fe}_{2}O_{3}$$

## Figures and Tables

**Table 1:** Water quality of desulfurization wastewater.

Projects	Numerical Value
pH	6.53
Conductivity, μS/cm	36,200
Turbidity, NTU	24.3
Tlkalinity, mmol/L	10.51
Stiffness, mmol/L	258.00
1/2Ca^2+^	39.80
1/2Mg^2+^	218.20

**Table 2:** Electrochemical parameters of Q235-A steel in L_0.1_–L_3_.

Lable	I_corr_ (A/cm^2^)	E_corr_ (A/cm^2^)
L_0.1_	1.750 × 10^−5^	−0.7226
L_0.3_	2.497 × 10^−5^	−0.7339
L_0.5_	3.993 × 10^−5^	−0.7138
L_1_	3.897 × 10^−5^	−0.7256
L_2_	3.614 × 10^−5^	−0.7184
L_3_	2.497 × 10^−5^	−0.7252

**Table 3:** Electrochemical parameters of the fitted corrosion product film.

Solution (×10^3^ mg/L)	Time (d)	R_s_ (Ω·cm^2^)	CPE_1_10^−4^ (Ω^−1^ cm^−2^S^−n^)	n_1_	R_p_ (Ω·cm^2^)	CPE_2_10^−4^ (Ω^−1^ cm^−2^S^−n^)	n_2_	R_ct_ (Ω·cm^2^)
1000	0 3 5	53.66 54.63 58.00	26.61 16.10 7.559	0.3998 0.6985 0.5055	196.8 271.2 540.4	266.1 502.6 105.32	0.7391 0.6977 1.000	712 717.4 1198
3000	0 3 5	19.21 21.51 19.95	3.275 5.564 3.864	0.7779 0.7567 0.7836	54.92 149.05 213.6	48.77 129.6 87.59	0.3768 0.3635 0.4189	500.1 533.8 556.1
5000	0 3 5	12.57 12.12 16.17	5.109 2.053 8.191	0.8690 0.8491 0.7851	38.86 50.22 248.6	36.61 38.00 31.45	0.6776 0.5417 0.9327	486.4 484.8 499.5
10,000	0 3 5	8.643 9.286 11.42	3.320 4.146 9.929	0.4271 0.4200 0.6604	38.43 26.16 13.74	22.08 19.62 22.25	0.8950 0.9126 0.9893	239.0 108.9 64.97
20,000	0 3 5	6.340 4.300 5.673	5.564 4.017 6.145	0.7567 0.9204 0.8225	149.0 132.3 112.3	129.6 26.90 25.88	0.3635 0.5988 0.7317	362.0 342.4 266.8
Solution (×10^3^ mg/L)	Time (d)	R_s_ (Ω·cm^2^)	CPE_1_10^−4^ (Ω^−1^cm^−2^S^−n^)	n_1_	R_p_ (Ω·cm^2^)	CPE_2_10^−4^ (Ω^−1^cm^−2^S^−n^)	n_2_	R_ct_ (Ω·cm^2^)
30,000	0 3 5	4.173 3.980 5.126	8.575 8.907 2.860	0.4747 0.8012 0.8903	240.1 179.6 136.3	43.93 25.08 19.62	0.5182 0.7259 0.9126	336.8 273.3 256.9

## Data Availability

Data cannot be disclosed due to privacy requirements.
